# Exploring Toxicity of Per- and Polyfluoroalkyl Substances (PFAS) Mixture Through ADMET and Toxicogenomic In Silico Analysis: Molecular Insights

**DOI:** 10.3390/ijms252212333

**Published:** 2024-11-17

**Authors:** Katarina Baralić, Teodora Petkovski, Nađa Piletić, Đurđica Marić, Aleksandra Buha Djordjevic, Biljana Antonijević, Danijela Đukić-Ćosić

**Affiliations:** Department of Toxicology “Akademik Danilo Soldatović”, Faculty of Pharmacy, University of Belgrade, Vojvode Stepe 450, 11221 Belgrade, Serbia; tpetkovksi@gmail.com (T.P.); pileticnadja8@gmail.com (N.P.); djurdjica.maric@pharmacy.bg.ac.rs (Đ.M.); aleksandra@pharmacy.bg.ac.rs (A.B.D.); biljana.antonijevic@pharmacy.bg.ac.rs (B.A.); danijela.djukic.cosic@pharmacy.bg.ac.rs (D.Đ.-Ć.)

**Keywords:** PFAS, genes, toxicokinetics, perfluorooctanesulfonic acid, perfluorooctanoic acid, perfluorohexanesulfonic acid, perfluoro-nonanoic acid, perfluorodecanoic acid, perfluoroundecanoic acid, perfluoroheptanesulfonic acid

## Abstract

This study aimed to explore the health impacts, mechanisms of toxicity, and key gene biomarkers of a mixture of the most prominent perfluoroalkyl/polyfluoroalkyl substances (PFAS) through in silico ADMET and toxicogenomic analysis. The following databases and tools were used: AdmetSAR (2.0), ADMETlab (2.0), Comparative Toxicogenomic Database, ToppGene Suite portal, Metascape (3.5), GeneMANIA server, and CytoHubba and CytoNCA Cytoscape (3.10.3) plug-ins. ADMET analysis showed that PFAS compounds pose risks of organ-specific toxicity, prolonged retention, and metabolic disruptions. Forty mutual genes were identified for all the tested PFAS. The mutual gene set was linked to disruption of lipid metabolism, particularly through nuclear receptors. The most important gene clusters identified were nuclear receptor signaling and PPAR signaling pathways, with kidney and liver diseases, diabetes, and obesity as the most significant related diseases. Phenotype data showed that PFAS compounds impact cell death, growth, inflammation, steroid biosynthesis, and thyroid hormone metabolism. Gene network analysis revealed that 52% of the 40 mutual genes showed co-expression, with co-localization as the next major interaction (18.23%). Eight key genes were extracted from the network: *EHHADH*, *APOA2*, *MBL2*, *SULT2A1*, *FABP1*, *PPARA*, *PCK2*, and *PLIN2*. These results highlight the need for further research to fully understand the health risks of PFAS mixtures.

## 1. Introduction

Per- and polyfluoroalkyl substances (PFAS) are synthetic compounds known for their chemical stability, which results in their long-term persistence in the environment [1]. These substances exhibit high mobility, making them prevalent even in remote regions [2]. Because these substances accumulate in both animal and human organisms, while their concentration rises as they move up the food chain, they are often called “forever chemicals” [3]. PFAS are known for their resistance to stains, heat, oil, grease, and water. This makes them highly valuable in a range of applications: these substances prevent food from sticking to cookware, enhance stain resistance in furniture and carpets, provide waterproofing for clothing and mattresses, and ensure grease resistance in certain food packaging materials [4]. Moreover, some of the most common applications of these substances include the synthesis of pesticides, production of firefighting foams, automobiles, cosmetics, the aviation and textile industry, etc. [5,6,7]. It is widely accepted that the consumption of food and drinking water is the main route of exposure for the general population to these substances [8,9], while inhalation and dermal contact are significant for occupational toxicology [10]. PFAS oral absorption is ranging from over 50% to above 95%, and these substances are extensively distributed in the liver, kidneys, and blood, where they bind to albumin and other proteins. They are not metabolized and are primarily eliminated through urine, with smaller quantities found in feces and breast milk [11]. PFAS toxicity primarily arises from activating peroxisome proliferator-activated receptor alpha (PPARα), impacting lipid metabolism [12]. It has been suggested that PPARα activation by these substances differs greatly depending on the functional group, length of the carbon chain, and species of the receptor [13]. PFAS toxicity mechanisms also involve interactions with other nuclear receptors, such as constitutive androstane receptor (CAR), pregnane X receptor (PXR), and possibly farnesoid X receptor (FXR) [12]. Consequently, these substances are associated with many harmful effects on human health, including hormonal imbalance, immune system dysfunction, liver damage, growth and developmental disorders, reproductive issues, and the development of cancer [14]. The two most well-known and extensively studied PFAS compounds are perfluorooctanoic acid (PFOA) and perfluorooctanesulfonic acid (PFOS), as documented in the 2018 report by the Agency for Toxic Substances and Disease Registry (ATSDR). In response to growing concerns about the harmful effects of PFOA on human health, wildlife, and the environment, the U.S. Environmental Protection Agency (EPA) launched the PFOA Stewardship Program in 2006 [15]. According to the assessment by the European Food Safety Authority (EFSA), the seven most prominent PFAS compounds in terms of human exposure include PFOS, PFOA, perfluorohexanesulfonic acid (PFHxS), perfluoro-nonanoic acid (PFNA), perfluorodecanoic acid (PFDA), perfluoroundecanoic acid (PFUnDA), and perfluoroheptanesulfonic acid (PFHpS) [16].

Due to their environmental persistence and potential harm to human health, the Stockholm Convention lists several PFAS, with an aim to restrict (Anex B) or eliminate the production and use of these chemicals (Anex A). In 2009, PFOS, its salts, and perfluorooctane sulfonyl fluoride (PFOSF) were included in Annex B, with amendments in 2019 to restrict exemptions. PFOA, its salts, and related compounds were added to Annex A in 2019, and PFHxS, its salts, and related compounds were included in 2022. The review of long-chain perfluorocarboxylic acids (LC-PFCAs) for potential listing is ongoing [17].

Nevertheless, despite these restrictions, the widespread nature of these substances and their pervasiveness ensure their continued existence in our environment. However, regardless of the fact that people are exposed to PFAS in mixtures [16], there is a scarcity of mechanistic toxicity studies on PFAS mixtures, which could offer valuable insights into how exposure to multiple PFAS compounds is linked to adverse health effects. In vitro studies on PFAS mixtures have shown that these compounds can produce additive effects or interact in synergistic or antagonistic ways. The type of interaction depends on several factors, such as the species under investigation, the levels and ratios of doses, and the specific components of the mixture [13]. Regarding PPARα activation, synergistic effects were observed in PFAS mixtures containing PFOS, PFNA, and PFHxS when tested on an in vitro model of human hepatocytes [18]. Additionally, binary mixtures of PFOA with PFOS, PFHxA, PFNA, or PFHxS exhibited additive effects in PPARα activation at lower doses, while higher doses resulted in more pronounced, synergistic effects in transfected kidney cell line COS-1 [19,20]. Considerable PPARα activation was also demonstrated in a 12-week study on male and female C57BL/6J mice exposed to a mixture of five PFAS (PFOA, PFOS, PFNA, PFHxS, and hexafluoropropylene oxide dimer acid (GenX)) [21].

Artificial intelligence, a rapidly growing branch of computer science, increasingly uses various machine learning techniques to predict and assess the toxicity of chemicals [22]. Considerable efforts have gone into developing in silico models and leveraging toxicogenomics data to improve alternative methods for assessing human health risks [23].

Our environment contains various small molecular compounds—such as medications, pesticides, food additives, industrial chemicals, and pollutants—that impact our health. Assessing absorption, distribution, metabolism, excretion, and toxicity (ADMET) properties has become essential to evaluate their potential effects or risks to the human body [24]. On the other hand, toxicogenomics enables the effective identification of health risks by analyzing gene–environmental stressors’ interactions in disease development [25,26], thus enabling the prediction of relevant biological interactions [27]. In mechanistic toxicology, toxicogenomics is suitable for (a) identifying gene ontology (biological processes, molecular functions, cellular components), molecular pathways and connected diseases based on the input gene sets, and (b) discovering genomic biomarkers linked to chemical exposure [28,29]. Additionally, in the context of studying the toxicity of mixtures, toxicogenomics aims to uncover mutual pathways and interactions where chemicals might act together, leading to possible additive or synergistic effects. This approach allows for a comprehensive assessment of how various chemicals interact with genes, revealing potential combined effects within a mixture [25]. One of the conducted toxicogenomic studies which assessed the link between PFAS mixtures and polycystic ovary syndrome (PCOS) found 74 genes linked to both PFAS exposure and PCOS, highlighting cell cycle regulation and steroid hormone synthesis—particularly genes like *CCNB1* and *SRD5A1* [30]. However, despite the critical need for research, in silico toxicogenomic studies assessing the overall toxicity potential of PFAS mixtures (especially one of the most prominent PFAS) remain notably scarce.

To address this gap, the current research aims to perform an in silico ADMET analysis to evaluate the toxicokinetics and toxicity of a PFAS mixture, with a focus on shared ADMET properties. Following this, toxicogenomic data mining will be performed to investigate the gene-level mechanisms driving the observed toxic effects.

## 2. Results

### 2.1. ADMET Analyses

The results of the ADMET analysis performed using admetSAR are presented in Table 1, providing insights into absorption, bioavailability, transporter inhibition, enzyme inhibition, receptor binding, metabolism and toxicity endpoints. A “+” indicates that the substance is associated with the respective ADMET property, while a “−” indicates no association according to the predictions.

Additionally, more detailed findings from the ADMETlab analysis are summarized in Supplementary Table S1.

### 2.2. Toxicogenomic Analyses

In the CTD database, out of the seven examined PFAS, PFHpS showed interactions with only one gene, *HSD11B2*, which was not found on the common gene list for the remaining six substances. Bearing this in mind, PFHpS was excluded from further investigation. For the remaining 6 PFAS compounds, a set of 40 mutual genes was identified: *A2M*, *ABCA1*, *ACOT2*, *ALB*, *ANGPTL4*, *APOA2*, *CPT1A*, *CYP2B10*, *CYP3A11*, *CYP7A1*, *DDIT3*, *EHHADH*, *ESR1*, *FABP1*, *FAS*, *FASN*, *GSTM3*, *HADHA*, *HGF*, *HMGCR*, *HMGCS1*, *HMGCS2*, *HMOX1*, *IL6*, *MBL2*, *NFE2L2*, *NR1I2*, *PCK2*, *PDK4*, *PLIN2*, *POR*, *PPARA*, *PPARG*, *PTPN11*, *SERPIND1*, *SLCO1A1*, *SOD1*, *SULT2A1*, *TTR*, *TXNIP*.

Full names of each of these genes are given in the Supplementary File S1 Table S2.

Forty mutual genes were used for ToppGene Suite analysis to provide insights into how these genes are functionally connected and biologically relevant in the context of PFAS effects. Table 2 provides a detailed overview of the extracted molecular functions, biological processes, molecular pathways, and diseases identified for the 40 mutual genes extracted from the 6 examined PFAS substances. This table highlights the shared mechanisms and pathways implicated by these substances, offering insights into their collective biological impact.

**Figure 1:**
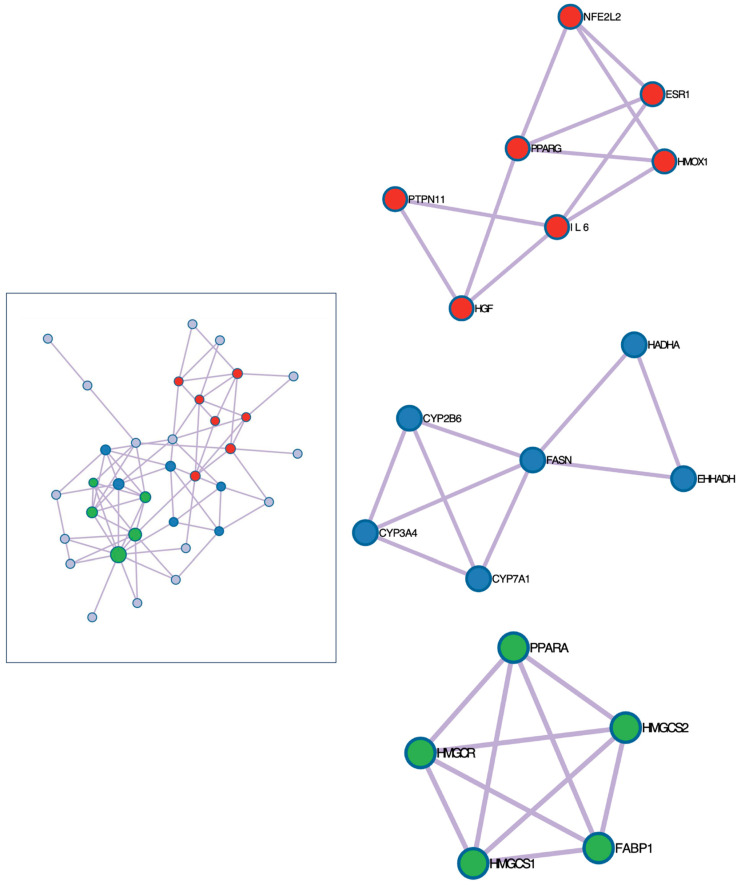
Interconnected gene clusters involved in the mechanism of toxicity of PFAS chemicals obtained by MCODE analysis (Metascape 3.5 software; https://metascape.org; accessed on 16 August 2024). Colors of the nods represent different clusters. Red cluster: pathways related to cancer and lung fibrosis; Blue cluster: related to liver X receptor pathway, monocarboxylic acid metabolic process and carboxylic acid metabolic process; Green cluster: related to PPAR signaling pathway.

The clusters generated using *MCODE* analysis are shown in Figure 1. As depicted in the figure, three interconnected gene clusters were detected. The identified MCODE networks are associated with significant biological processes and pathways (Table 3). The Red network is linked to pathways involved in cancer and lung fibrosis. The Blue network is connected to the Liver X receptor pathway and metabolic processes involving carboxylic acids. The Green network is related to the regulation of lipid metabolism and the PPAR signaling pathway. The most relevant gene ontology processes obtained by MCODE analysis and connected to the three identified clusters were nuclear receptors’ meta pathway, when *PPARA* activates gene expression, and regulation of lipid metabolism by PPARalpha.

Eight common phenotypes were found for all the investigated PFAS: cell death, cell growth, cell population proliferation, inflammatory response, positive regulation of cell population proliferation, positive regulation of telomere maintenance via telomere lengthening, the steroid biosynthetic process, and the thyroid hormone metabolic process (Supplementary Table S2). The phenotypic data identified in the CTD were derived from in vitro and in vivo studies conducted on three organisms—rats, mice, and zebrafish—as well as human biomonitoring studies. Supplementary File S2 contains the detailed data about the phenotype–gene interactions.

**Figure 2:**
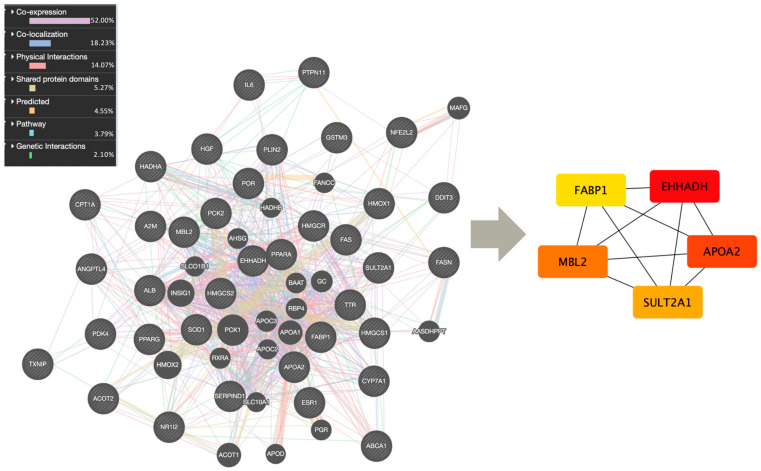
Interactions between the genes associated with exposure to PFAS mixtures (GeneMANIA (https://genemania.org); accessed on 15 August 2024) and the 5 hub genes identified (CytoHubba Cytoscape; accessed on 15 August 2024).

Next, network analysis was conducted on the 40 mutual genes to examine their interactions and identify key genes that serve as central hubs or connectors within the network. Over half of these genes exhibited co-expression (52.00%), with co-localization representing the second most significant form of interaction among them (18.23%) (Figure 2).

For a clearer understanding of the specific relationships within each interaction type, Supplementary Figure S1 in Supplementary File S1 presents the GeneMania network, showing both a combined interaction view and separate circular layouts for individual interaction types.

Among the gene network, five genes were identified as hub genes: *EHHADH*, *APOA2*, *MBL2*, *SULT2A1*, and *FABP1*.

Further centrality analysis highlighted key genes in the network that have strong interactions with others and play significant roles in connecting different pathways (Supplementary Table S3 in File S1). The genes *FABP1*, *EHHADH*, *APOA2*, *PPARA*, and *PCK2* show the highest degree and closeness, indicating they are central hubs with extensive interactions and the capacity to quickly influence other nodes. Additionally, *FABP1*, *EHHADH*, *APOA2*, *PCK2*, and *PLIN2* had the highest betweenness, suggesting they are key connectors within the network. These combined metrics highlight their critical roles in maintaining network connectivity and overall regulatory influence.

For each PFAS chemical examined in the study, relevant chemical–hub gene interaction data were extracted from the CTD (*EHHADH*, *APOA2*, *MBL2*, *SULT2A1*, *FABP1*). Three additional relevant genes (*PPARA*, *PCK2*, and *PLIN2*) obtained by centrality analysis were also taken into account. The analysis focused on identifying the most significant interactions, with particular emphasis on mRNA expression (ME) and protein expression (PE) changes. These findings are detailed in Table 4.

The upregulated and downregulated key genes were compiled and analyzed using ToppGene Suite to further explore the potential biological implications of PFAS effects through systems biology analyses (Supplementary File S1, Tables S4 and S5). The upregulated genes (*EHHADH*, *APOA2*, *FABP1*, *PPARA*, *PCK2*, and *PLIN2*) were linked to various molecular functions, including lysophospholipid symporter activity, phosphoenolpyruvate carboxykinase activity, phosphoenolpyruvate carboxykinase (GTP) activity, long-chain-3-hydroxyacyl-CoA dehydrogenase activity, and high-density lipoprotein particle receptor binding. Additionally, these genes were associated with biological processes such as cellular lipid catabolic processes, lipid catabolic processes, fatty acid catabolic processes, monocarboxylic acid catabolic processes, and positive regulation of lipid catabolic processes. The analysis also revealed connections to key pathways, including the PPAR signaling pathway and PPAR-alpha pathway, as well as diseases such as acute kidney tubular necrosis, chronic kidney failure, fatty liver, steatohepatitis, and hypertensive encephalopathy. The downregulated genes (*MBL2*, *SULT2A1*, *FABP1*, *PPARA*), mostly downregulated by PFDA and/or PFUnDa, were linked to molecular functions such as glycochenodeoxycholate sulfotransferase activity, lysophospholipid symporter activity, lipid binding, bile-salt sulfotransferase activity, and alcohol sulfotransferase activity. These downregulated genes were associated with biological processes including monocarboxylic acid catabolic processes, positive regulation of fatty acid beta-oxidation, positive regulation of fatty acid oxidation, regulation of fatty acid beta-oxidation, and carboxylic acid catabolic processes. Furthermore, they were connected to key pathways like the regulation of lipid metabolism by PPAR-alpha, the PPAR-alpha pathway, and the nuclear receptors meta pathway, as well as diseases such as acute kidney tubular necrosis, chronic kidney failure, cystic fibrosis, Alzheimer’s disease, and mannose-binding protein deficiency.

## 3. Discussion

Since PFAS often contaminate soil, groundwater, and drinking water, leading to exposure to multiple compounds, it is essential to assess their combined effects to fully understand their impact on human health [31]. Considering this, several in vitro and animal studies have explored the toxicity of their mixtures. One of the in vitro studies aimed to investigate how PFAS mixtures composed of PFOS, PFOA, PFNA, and PFHxS affect lipid metabolism in human HepaRG cells, a model for human hepatocytes. The authors demonstrated that PFAS induced triglyceride accumulation and altered the expression of genes associated with steatosis, PPARα target genes, and genes related to lipid and cholesterol metabolism [18]. Furthermore, binary mixtures of PFOA with PFOS, PFHxA, PFNA, or PFHxS showed additive effects on PPARα activation at lower doses, whereas higher doses led to more significant, synergistic effects in transfected COS-1 cells [19,20]. In a 12-week in vivo study, male and female C57BL/6J mice exposed to a mixture of five PFAS (PFOA, PFOS, PFNA, PFHxS, and GenX) at 2 mg/L showed significant PPARα activation and elevated serum cholesterol levels. The results have also shown increased sterol metabolites and bile acids, along with hepatic injury, inflammation, and elevated alanine aminotransferase levels [21]. Furthermore, after the exposure of male C57BL/6J mice to a mixture of nine PFAS (PFOA, perfluorobutanoic acid (PFBA), PFOS, perfluoropentanoic acid (PFPeA), PFNA, PFHxA, PFBS, PFHpA, and PFHxS) at concentrations mirroring human daily intake (1 µg/L and 50 µg/L) for five weeks, morphological changes in the liver were observed, such as hepatocyte vacuolization and irregular hepatocyte cord arrangement. Additionally, alterations in genes associated with metabolism and carcinogenesis were reported, as well as changes in substances like glutathione and 5-aminovaleric acid, suggesting significant impacts on liver morphology and metabolic processes [32]. After the exposure of male C57BL/6 mice to a PFAS mixture (PFOS, PFOA, PFNA, PFHxS, GenX) through drinking water at a concentration of 20 µg/L for 18 weeks, researchers found significant changes in sperm methylation and altered gene expression in the liver and fat of male offspring, affecting cholesterol metabolism, cell cycle regulation, and myeloid leukocyte migration. In females, PFAS exposure impacted erythrocyte development and carbohydrate metabolism [33]. All of these studies highlighted the urgent necessity to further comprehend the collective impacts of PFAS exposure. However, to the best of our knowledge, a mixture of the most prominent PFAS chemicals has not yet been assessed in vitro, in vivo, or in silico. Hence, we aimed to conduct an in silico ADMET analysis to assess the toxicokinetics and toxicity of this PFAS mixture, followed by toxicogenomic data mining to explore the gene-level mechanisms driving these effects.

### 3.1. ADMET Analysis

Results of both admetSAR and ADMETlab analysis have highlighted potential health risks associated with PFAS substances, including potential for organ-specific toxicities, metabolic interference, and prolonged presence in the body. The results of admetSAR testing shown in Table 1 indicated a series of common characteristics for all tested substances, suggesting the potential for additive or synergistic effects when these substances are present in a mixture. According to these results, PFAS substances have shown a potential ability for intestinal absorption, contributing to their further distribution in the body. It is also suggested that all of these substances can pass through the blood–brain barrier, indicating their ability to reach the central nervous system. They were found to affect PPAR-gamma receptors, which promote fat storage and regulate hormones that maintain glucose homeostasis [34]. Additionally, all tested substances were associated with eye and skin irritation and damage, binding to estrogen receptors, nephrotoxicity, and carcinogenicity. On the other hand, the results of ADMETlab testing (Supplementary Table S1) have shown that, according to the Lipinski Rule [35], all substances meet the criteria, indicating they could be absorbed if ingested, leading to systemic exposure. Among these, PFHxS stood out with a higher potential for CNS toxicity. Furthermore, these results have indicated that all the tested PFAS have a prolonged presence in the bloodstream (high plasma protein binding (PPB)). Next, all of these compounds were identified as substrates for CYP1A2 and CYP3A4, enzymes responsible for drug metabolism [36]. Additionally, they were found to inhibit CYP2C8, and all except PFHxS also inhibited CYP2C9. Similarly, all PFAS, apart from PFHxS, were connected with p53, which is important for tumor suppression [37]. Toxicologically, these PFAS substances were found to potentially exhibit adverse effects, including eye corrosion and irritation, skin sensitization, respiratory toxicity, hepatotoxicity, nephrotoxicity, and genotoxicity.

### 3.2. Gene Ontology, Molecular Pathways and Related Diseases

To identify which of these predictions could stem from PFAS influence at the gene level and to explore the specific molecular mechanisms, we conducted toxicogenomic data mining. Forty genes shared across all the examined PFAS substances were identified. When considering all 40 mutual genes obtained by toxicogenomic analysis together, associated molecular functions, biological processes, and molecular pathways were identified to explore potential molecular mechanisms of PFAS toxicity. The list of extracted gene ontology terms and pathways can be broadly grouped into categories related to lipid metabolism, nuclear receptor activity, and responses to various stimuli. Lipid metabolism encompasses processes like cholesterol metabolism, fatty acid oxidation, and sterol and steroid metabolism, as seen in terms like hydroxymethylglutaryl-CoA synthase activity, oxidoreductase activity, acyltransferase activity, and pathways such as PPAR signaling and *SREBF* and *MIR33* in cholesterol and lipid homeostasis. MiR-33, found within the intron of sterol regulatory element-binding protein (SREBP) 2, plays a role in regulating cholesterol homeostasis [38], while the nuclear hormone receptor (NHR) superfamily comprises transcriptional regulators crucial for pathways like development, growth, and metabolism. Activated by ligands like hormones, these receptors regulate gene expression [39]. The response to stimuli category includes terms like response to nutrient levels, response to xenobiotic stimulus, and cellular response to an oxygen-containing compound, often mediated by nuclear receptors and related pathways. These categories are mutually connected, particularly through the regulation of lipid metabolism by nuclear receptors such as *PPARA*. Nuclear receptors and PPAR signaling pathways were also identified as particularly important clusters in MCODE analysis. *PPARA* is a gene which plays a crucial role in regulating lipid metabolism and uptake [40]. As seen in Figure 2, Green identifies the network’s association with the regulation of lipid metabolism, and the PPAR signaling pathway highlights the impact of PFAS on lipid homeostasis, potentially contributing to disorders such as dyslipidemia or fatty liver disease. The most relevant gene ontology processes identified—nuclear receptors’ meta pathway, *PPARA* activation, and regulation of lipid metabolism by PPARalpha—further emphasize the role of PFAS in disrupting key metabolic and regulatory pathways. The Red network’s connection to pathways involved in cancer and lung fibrosis suggests that PFAS exposure may contribute to or exacerbate these health conditions. The Blue network, which is linked to the Liver X receptor pathway and various carboxylic acid metabolic processes, highlights potential disruptions in metabolic regulation and homeostasis. The liver X receptor pathway influences genes responsible for proteins that manage cholesterol absorption, transport, removal, excretion, and conversion to bile acids. Previously conducted toxicological studies link PFAS exposure to various health risks, including developmental, reproductive, neuro-, hepato-, and immunotoxicity, as well as thyroid disruption and cancer [41]. Effects on growth and development have been observed in children, including changes in behavior or early puberty onset, as well as in newborns, where reduced birth weight has been noted [42]. Long-term exposure to PFAS in the general population is associated with an increased risk of kidney, prostate, and testicular cancer, along with disturbances in cholesterol metabolism and reduced effectiveness of the immune system against infections [42]. However, the current toxicogenomic study has shown that the most significant diseases associated with exposure to the investigated mixture of PFAS include kidney dysfunction (acute kidney injury, acute kidney damage, chronic kidney insufficiency, Bright’s disease (nephritis)) and liver disease (drug-induced liver disease, acute drug-induced liver damage, liver damage caused by chemicals and drugs, hepatitis caused by toxic substances, etc.) and the development of diabetes/obesity, which may indicate the interrelated effects that may occur after exposure to all these substances simultaneously.

### 3.3. Phenotype Data

Phenotype data was explored and downloaded from the CTD to investigate the direct link between molecular-level changes and observable biological outcomes. Eight common phenotypes were found for all the investigated PFAS: cell death, cell growth, cell population proliferation, inflammatory response, positive regulation of cell population proliferation, positive regulation of telomere maintenance via telomere lengthening, the steroid biosynthetic process, and the thyroid hormone metabolic process. Phenotype analysis also revealed dual interactions (Supplementary File S2), suggesting that the effect is influenced by variables such as study type (in vitro, in vivo, human), species, cell type, concentration, and overall study design. For example, PFOS was found to both increase and decrease cell death, depending on the type of cells it was investigated in and whether it was present alone or in mixture with other PFAS. This substance increased cell death on non-tumor human hepatic cells (L-02) in concentrations of 25 and 50 mg/L [43], while it decreased cell death (U2OS-GR cells) when present in a mixture with other PFAS substances [44]. Similarly, PFOS led to both increased and decreased cell growth. When investigated in vivo, this substance increased cell growth in mice livers after exposure to 5 mg/kg/day, 28 days, per os On the other hand, it decreased cell growth in human lung A549 cells (concentrations 25, 50, 100 and 200 μM) [45] and rat neurons (concentration 60 μM) [46]. Likewise, PFOA could both increase and decrease cell death. When present alone, it was found to increase cell death in zebrafish liver cell line (0.5, 5, or 10 ppm) [47], rat mast cells (100 and 500 μM) [48], and human trophoblasts (0.1, 1, 10, 25, 100, or 250 µM) [49], while it decreased cell death in the mixture with other PFAS in U2OS-GR cells [44]. Furthermore, phenotype data suggested that PFAS might decrease the inflammatory response when examined individually. However, when PFOS is co-treated with another compound (e.g., 3,4,5,3′,4′-pentachlorobiphenyl (a type of polychlorinated biphenyl), it increased the inflammatory response [50]. Similarly, the data have suggested that all the examined PFAS affected the steroid biosynthetic process and the thyroid hormone metabolism. However, PFOS was found to be connected to both decreased and increased thyroid hormone metabolic processes, resulting in altered secretion of thyroxine (T4) and triiodothyronine (T3), while iodine deficiency was found to exacerbate the effects of some PFAS compounds. This data was derived from a human cross-sectional study involving 1525 adults, which demonstrated that PFHxS and PFOS showed a negative correlation with free thyroxine (fT4), while all four measured PFAS compounds (PFHxS, PFNA, PFOA, PFOS) were positively correlated with free triiodothyronine (fT3), the fT3/fT4 ratio, thyroid stimulating hormone (TSH), and total triiodothyronine (TT3) in the group with combined high thyroid peroxidase antibodies (TPOAb) levels and low iodine [51]. Other phenotype data in the CTD was also derived from human biomonitoring studies, allowing the correlation of PFAS environmental exposure levels with specific biological outcomes. One of these studies analyzed serum and umbilical cord samples from 84 pregnant women to assess effects on metabolic processes. PFNA had the lowest concentration linked to steroid biosynthesis in serum (geometric mean: 0.90 ng/mL). Expectedly, fetal exposure to PFAS was generally lower than adult exposure. For instance, geometric mean fetal concentration of PFOS was 2.53 ng/mL, compared to 5.36 ng/mL in adults. Similar trends were seen with other PFAS (PFDA: adult: 0.87 ng/mL, fetus: 0.37 ng/mL). However, despite reduced fetal exposure, these compounds were still found to impact steroid biosynthesis [52]. Another study showed that PFOS in maternal blood significantly affected fetal thyroid hormone metabolism (300 mother-infant pairs, PFOS geometric mean concentration in mothers’ blood: 10.77 ng/mL) [53]. PFOA impacted the inflammatory response at median plasma concentrations of 3.31 ng/mL [54], while this substance affected telomere maintenance in fetal blood even at lower concentrations (0.8 ng/mL) [55].

### 3.4. Network Analysis

Following the construction of a network with the previously identified 40 shared genes, co-expression—observed in 52% of these genes—indicated similar expression patterns, suggesting functional relationships or involvement in shared biological processes. Co-localization, observed in 18.23% of the genes, involves genes expressed in the same tissue or proteins located in the same cellular area, indicating they may work together or have related functions. In the centrality analysis, genes such as *FABP1*, *EHHADH*, *APOA2*, *PPARA*, and *PCK2* were identified as central hubs within a biological interaction network due to their high degree and closeness centrality metrics, which means they have numerous connections with other genes and are strategically positioned to rapidly influence the activity of neighboring nodes. Additionally, *FABP1*, *EHHADH*, *APOA2*, *PCK2*, and *PLIN2* had the highest betweenness, suggesting they are key connectors within the network, facilitating communication between different parts of the system.

Hence, after considering centrality and hub gene analysis, *EHHADH*, *APOA2*, *MBL2*, *SULT2A1*, *FABP1*, *PPARA*, *PCK2*, and *PLIN2* were marked as the most significant within the set of 40 common genes. *EHHADH* encodes an enzyme that is involved in beta-oxidation of fatty acids [56]. *APOA2* is a gene that encodes a protein crucial for lipid metabolism, particularly in the transport and regulation of cholesterol levels [57,58]. *MBL2* is the gene responsible for producing the lectin protein that binds to mannose, which plays a role in the immune response [59]. *SULT2A1* encodes an enzyme which facilitates the sulfate conjugation of dehydroepiandrosterone (DHEA) and various other steroids [60], while *FABP1* is the gene that encodes a protein bound to fatty acids and plays a key role in the transport of fatty acids [57,61]. *PPARA* regulates lipid metabolism and fatty acid oxidation [62], *PCK2* is essential for gluconeogenesis and glucose production [63], and *PLIN2* modulates lipid storage and mobilization by coating lipid droplets [64].

Each of these genes contributes to various aspects of metabolism and organismal function, which could potentially be disrupted by PFAS. These 8 key genes underwent further examination in the CTD for specific interactions with PFAS compounds. All PFAS, apart from PFNA, increased *EHHADH* mRNA expression. PFNA exhibited a variable effect, meaning it could either increase or decrease *EHHADH* mRNA. All PFAS increased PCK2 mRNA expression, and all PFAS increased PLIN2 mRNA expression, apart from PFOS and PFOA, which could both increase and decrease it. PFOA, PFHxS, and PFNA elevated *APOA2* mRNA expression, whereas PFDA and PFUnDA showed variable effects, causing both an increase and decrease. In the case of the other three hub genes, PFAS compounds elicited varied responses, with some increasing mRNA gene expression and some decreasing it, while others displayed a dual effect. PFDA and PFUnDA demonstrated opposing effects on certain genes compared to other PFAS, as these substances downregulated some of the genes that other PFAS were found to upregulate (*MBL2*, *SULT2A1*, *FABP1*, and *PPARA*).

The upregulated genes identified from the analysis of PFAS, specifically *EHHADH*, *APOA2*, *FABP1*, *PPARA*, *PCK2*, and *PLIN2*, play crucial roles in lipid metabolism and homeostasis. ToppGene enrichment analysis for these genes is given in the Supplementary File S1, Tables S4 and S5. There, according to the top 5 extracted molecular functions, biological processes, pathways, and diseases, it can be concluded that associated molecular functions, including lysophospholipid symporter activity and long-chain-3-hydroxyacyl-CoA dehydrogenase activity, suggest a heightened capacity for lipid processing and transport, aligning with the biological processes linked to lipid catabolism and cellular lipid degradation. These genes are implicated in significant pathways, such as the PPAR signaling pathway. The connections to diseases such as acute kidney tubular necrosis and chronic kidney failure further highlight the potential pathophysiological implications of PFAS exposure, indicating that dysregulation of lipid metabolism may contribute to the development of conditions like fatty liver and steatohepatitis. The downregulated genes *MBL2*, *SULT2A1*, *FABP1*, and *PPARA*, specifically in response to PFDA and PFUnDA exposure, also reveal significant linkage with lipid metabolism. Their associated molecular functions, such as glycochenodeoxycholate sulfotransferase activity and lipid binding, suggest an impaired ability to process lipids effectively, reflected in biological processes related to fatty acid catabolism and regulation. The involvement of these genes in critical pathways like the lipid metabolism regulation by PPARalpha indicates potential metabolic dysfunction. Furthermore, their links to diseases such as acute kidney tubular necrosis, chronic kidney failure, cystic fibrosis, and Alzheimer’s disease emphasize the broader health implications of PFAS exposure.

It is important to note that the metabolic context and specific PFAS involved play a critical role in determining whether a gene is upregulated or downregulated. The differences may also reflect variations in exposure levels, duration of exposure, and tissue-specific responses to the chemical stressors. Thus, while PFDA and PFUnDA lead to a downregulation of certain genes, other PFAS may enhance gene expression to promote lipid metabolism, highlighting the complexity of PFAS effects on biological systems.

### 3.5. Critical Endpoints

The results of the current ADMET and toxicogenomic study indicate that exposure to the investigated mixture of PFAS is associated with significant disruptions in biological pathways, critical endpoints, and diseases related to lipid metabolism, liver toxicity, kidney function, CNS toxicity, thyroid function disruption, and diabetes. These findings align with in vivo studies that demonstrate the effects of PFAS exposure. As already mentioned, our analysis revealed that lipid metabolism, particularly through nuclear receptors and the PPAR signaling pathway, is significantly disrupted by PFAS exposure. The study conducted by Elcombe et al. (2012) revealed that rats orally exposed to 20 or 100 ppm of PFOS for 7 days exhibited several changes in liver parameters. These included an increase in liver weight and the upregulation of specific enzymes such as acyl CoA oxidase, CYP4A, CYP2B, and CYP3A, which was also indicated in our ADMET predictions. The activation of PPAR and CAR/PXR pathways was identified as a key factor contributing to PFOS-induced liver enlargement and the potential for hepatic tumor formation [65]. Similarly, after treating male rats with PFOA for 5 days at varying doses, it was suggested that higher doses of 5 and 20 mg/kg/day downregulated genes related to fatty acid and steroid metabolism, indicating that longer-chain PFAS impair lipogenic pathways via inhibitory interactions between PPARα, PPARβ, and PPARγ [66]. In their bioinformatic approach, Yang et al. (2023) showed that the upregulation of hepatic acyl-CoA oxidase 1 (ACOX1) in the PPARα-regulated peroxisomal β-oxidation pathway was the key event linked to disrupted hepatic lipid metabolism caused by PFOA and PFOS in humans, mice, and rats. They later confirmed in vivo that administering PFOA and PFOS to mice at 1 mg/kg body weight for 35 days resulted in ACOX1-mediated oxidative stress, mitochondrial dysfunction, and lipid accumulation in hepatocytes [67]. These findings correlate with our MCODE analysis, which identified the involvement of lipid metabolism pathways and *PPAR* signaling as particularly important in PFAS mixture toxicity. Additionally, a study by Li et al. (2021) found that PFOS exposure in C57BL/6 mice at 10 mg PFOS/kg b.w./day by oral gavage for 14 days dysregulated proteins involved in lipid and xenobiotic metabolism, leading to liver morphological damage. The upregulation of ceramide and lysophosphatidylcholine (LPC), which induced liver cell apoptosis, further aligns with our findings of cell deaths-related gene interactions and phenotype [68]. Another study by Li et al. (2021) observed PFOS accumulation in the liver, lungs, kidneys, spleen, heart, and brain of BALB/c mice after exposure to 100 μg/kg b.w./day and 1000 μg/kg b.w./day for 2 months, causing damage in the liver, affecting glycerophospholipid metabolism and sphingolipid metabolism [69]. These in vivo studies support the hypothesis that PFAS exposure leads to disruptions in lipid metabolism, contributing to the development of liver diseases.

Next, our toxicogenomic analysis identified nephrotoxicity as another key endpoint. This aligns with the results from in vivo investigations, including the one carried out by Owumi et al. (2021), which observed elevated levels of renal biomarkers (urea and creatinine) in rats orally given 5 mg/kg b.w./day of PFOA for a duration of 28 days [70]. Furthermore, Rashid et al. (2020) observed renal damage in mice exposed to 1, 5, 10, or 20 mg/kg b.w./day of PFOA by oral gavage for 10 days, including increased expression of fibrotic markers (TGF-β and α-SMA) and hypermethylation of Rasal1, an early indicator of fibroblast activation [71]. Furthermore, in a study on PFOA exposure during pregnancy, 20 mice were gavaged 3.5 mg/kg of PFOA throughout the pregnancy period, which caused significant changes in kidney weight, histopathological alterations, and markers of oxidative stress. Transcriptomic analysis showed that PFOA disrupts kidney function in offspring by altering gene expression related to the circadian rhythm, the PPAR signaling pathway, and biosynthesis of unsaturated fatty acid [72].

Moreover, our study identified the potential for CNS toxicity, with evidence suggesting that PFAS may penetrate the blood–brain barrier and disrupt neuronal processes. Cui et al. (2009) reported that rats treated to 146 µg/b.w. of PFOS for 28 days had ten times higher brain concentrations than those exposed to lower doses, despite the higher dosage being just four times that of the lower group. The authors suggested that this phenomenon might be attributable to increased permeability of the blood–brain barrier at higher dosages, resulting in a larger accumulation of PFOS in the brain [73]. Sim and Lee (2022) documented long-term developmental neurotoxicity in mice exposed to 6.1 and 9.1 mg/kg b.w. PFHxS by oral gavage during neonatal exposure from postnatal day 10. This exposure led to memory impairment and the downregulation of neuronal proteins like GAP-43 and CaMKII [74]. These proteins are critical for synaptic plasticity and neuronal growth [75], and their downregulation aligns with our in silico findings of CNS toxicity.

Our findings also suggest that thyroid hormone metabolism may be affected by PFAS exposure, with potential implications for thyroid function. This is consistent with the study by Ramhøj et al. (2020), which found a dose-dependent decrease in thyroid hormone levels in rats exposed to 0.05, 5, or 25 mg/kg b.w./day PFHxS by oral gavage from gestation day 7 through to postnatal day 22 [76]. Additionally, after exposing adult male rats to 3 mg PFOS/kg/day for 7 days, decreased levels of T4 and T3 were observed [77]. The disruption of thyroid hormone levels in vivo, together with the extracted phenotype data, provides strong support for the pathway dysregulation observed in our gene set analysis, supporting the link between PFAS exposure and endocrine disruption.

A study investigated the effects of PFOS on pancreatic β-cell functions in mice orally treated with 1 and 5 μg/kg b.w./day for 21 days, revealing that PFOS treatment significantly increased liver triglycerides while decreasing glycogen levels, impaired insulin signaling pathways, reduced insulin and key transcription factor levels in pancreatic islets, and inhibited glucose-stimulated insulin secretion, thereby highlighting the molecular mechanisms by which PFOS may contribute to metabolic diseases [78], including diabetes, which was marked as another critical endpoint in our in silico investigation. Additionally, murine study reported elevated blood glucose levels and lower glycogen in the liver after exposure to 5 mg/kg b.w./day PFOA for 28 days. These authors reported increased phosphorylation of AKT and GSK3β after insulin stimulation in the livers of mice exposed to PFOA [79]. During mating, gestation, lactation, and up to 30 weeks of age, mice exposed to 3 μg/L of PFUnDA showed increased pancreatic insulitis, more apoptotic cells in pancreatic islets before insulitis, decreased peritoneal macrophage phagocytosis, fewer tissue-resident macrophages in pancreatic islets before insulitis, and changed cytokine secretion in activated splenocytes following exposure [80].

### 3.6. Limitations

Providing highly valuable insights into the effects of the tested PFAS mixture, our study highlights the effectiveness of freely accessible web-based tools for ADMET and toxicogenomic data analysis. This study demonstrates the value of toxicogenomic data mining for (i) developing hypotheses about links between chemical exposure and human diseases; (ii) pinpointing chemical effects on specific molecular targets and cellular pathways; (iii) predicting the combined toxicological effects of multiple substances; and (iv) extracting insights that can guide further in vitro and in vivo research. Additionally, ADMET analysis has proven useful in evaluating compounds’ pharmacokinetic and toxicity profiles, helping to predict absorption, distribution, metabolism, excretion, and potential toxicity.

However, it also reveals certain limitations associated with platforms such as the presented ADMET tools, CTD, ToppGene Suite, GeneMANIA, etc. These tools enhance traditional toxicity testing approaches by depending on the quality and thoroughness of online resources for their annotations. Challenges arise from issues such as incomplete interactions and the risk of false positives [81]. admetSAR and ADMETlab, while valuable tools for predicting ADMET properties, have several limitations. A key limitation of such tools is the quality and availability of data, as the accuracy of these models relies on high-quality experimental data for training [24]. The reliability of ADMET profiling predictions also relies on modeling tools utilized in model development. Consequently, issues such as experimental errors in the dataset, low-quality models, and the concept of applicability domain raise significant concerns about prediction accuracy [82]. The multifaceted nature of chemical exposure must also be considered; various factors—including dose, route of administration, exposure duration, metabolic processes, developmental stages, and a range of environmental conditions—significantly influence toxicity outcomes [83]. Interactions identified in the CTD can vary widely due to curation conditions and study design. Our results show that while some genes or proteins consistently respond to specific substances, others display both activation and inhibition, likely due to non-monotonic dose-response relationships, as often observed with endocrine disruptors [84]. In conclusion, while the in silico analysis described in our study provides important insights, additional laboratory studies are essential to investigate how chemical impacts on the identified gene sets can vary under different external conditions, such as dietary influences or methods of exposure.

## 4. Materials and Methods

In this study, a range of publicly available databases and software tools were used for in silico analysis. These included admetSAR (2.0) (https://lmmd.ecust.edu.cn/admetsar2; accessed on 11 August 2024), ADMETlab (2.0) (https://admetlab3.scbdd.com; accessed on 11 August 2024), Comparative Toxicogenomics Database (CTD; https://ctdbase.org; accessed on 15 August 2024), GeneMANIA (https://genemania.org; accessed on 15 August 2024 and 5 November 2024), CytoHubba (https://apps.cytoscape.org/apps/cytohubba; accessed on 15 August 2024) and CytoNCA (https://apps.cytoscape.org/apps/cytonca) Cytoscape (3.10.3) plug-ins (accessed on 15 August and 5 November 2024), ToppGene Suite portal (https://toppgene.cchmc.org; accessed on 16 August and 5 November 2024), and Metascape (3.5) (https://metascape.org; accessed on 16 August 2024). All data from these resources were downloaded in August and November 2024, ensuring the most up-to-date information was used. (These servers may occasionally be unavailable due to scheduled maintenance, upgrades, or data updates).

### 4.1. ADMET Properties: AdmetSAR and ADMETlab

To cross-validate the results and ensure a more comprehensive assessment, two ADMET tools were applied, each offering unique strengths and perspectives on the compound’s absorption, distribution, metabolism, excretion, and toxicity. AdmetSAR is a user-friendly tool for searching ADMET properties based on the structure of a specific compound. AdmetSAR contains a vast amount of information collected from various sources, enabling easy exploration of chemical profiles. This tool focuses on in silico prediction using computational models, facilitating the identification and assessment of compound safety [85]. Similarly, ADMETlab is an advanced platform focusing on evaluating ADMET chemicals’ properties. In its latest iteration, ADMETlab 3.0, it encompasses 119 ADMET endpoints, including 21 physicochemical, 20 medicinal chemistry, 9 absorption, 9 distribution, 14 metabolism, 2 excretion, 36 toxicity properties, and 8 toxicophore rules, offering a comprehensive array of assessments essential for informed decision-making [86].

### 4.2. Toxicogenomic Analysis

#### 4.2.1. Comparative Toxicogenomic Database

Comparative Toxicogenomic Database (CTD) is a public resource that evaluates links between chemicals, genes, and diseases, offering data on gene ontology, chemical exposure, phenotypes, etc. [87,88]. Regular updates are conducted to maintain the reliability, consistency, and accessibility of the information encompassed by this database [88].

In the CTD, data are collected from in vitro, animal, and human studies. However, because the CTD prioritizes environmental chemicals and their impact on human health, only those genes and proteins that exist in human body are included in the database [89]. CTD curators input chemical–gene interactions and disease associations into the CTD following guidelines set forth by a lead curator [90]. Before the curated data are publicly released on the CTD website, they are uploaded into a database for quality control assessment. Furthermore, quality control is enhanced by employing controlled vocabularies and ontologies to ensure consistency in the curated interactions [90,91]. As a result, the CTD utilizes official gene symbols and names from the National Center for Biotechnology Information’s (NCBI) Entrez-Gene database, while its disease vocabulary incorporates terms from MeSH (Medical Subject Headings) and OMIM (Online Mendelian Inheritance in Man) [83,88].

In this study, the CTD database was utilized to identify genes associated with exposure to PFAS substances. We used all the available data in the CTD, from both human and animal studies, as the animal studies contribute valuable insights into gene interactions and toxicological responses. We only collected curated data for our analysis, ensuring that all the information used was directly derived from peer-reviewed experimental studies without inferred interactions. As previously mentioned, EFSA identified seven of the most significant PFAS chemicals in the environment in terms of ubiquity and their impact on human health, which include PFOS, PFOA, PFHxS, PFNA, PFDA, PFUnDA, and PFHpS. Each of these chemicals was successfully located in the CTD database, and corresponding genes linked to these substances were identified. For the identification of the genes common to all seven examined PFAS substances, the MyVenn CTD tool (https://ctdbase.org/tools/myVenn.go; accessed on 15 August 2024) was utilized. Then, to obtain the mutual phenotypes associated with the investigated PFAS, VennViewer (https://ctdbase.org/tools/vennViewer.go; accessed on 15 August 2024) and MyVenn tools were used. Afterwards, data about each mutual phenotype were extracted for every PFAS, individually, from the phenotype data cards in CTD. Relevant dose/concentration levels were acquired from the original published literature available in CTD for each extracted phenotype interaction.

#### 4.2.2. ToppGene Suite Portal

The ToppGene Suite portal assesses gene functions. Its function, ToppFun, accessible at https://toppgene.cchmc.org/enrichment.jsp (accessed on 16 August and 5 November 2024), allows searching gene ontology, molecular pathways, phenotypes, microRNA interactions, and more [92]. This function was used to explore the potential molecular mechanisms associated with the combined effects of PFAS mixtures. A set of 40 common genes was used for the analysis, examining their association with biological processes, molecular functions, molecular pathways, and diseases. The significance level was determined based on a *p*-value of 0.05, with false discovery rate (FDR) correction.

#### 4.2.3. Metascape

Metascape(3.5) (https://metascape.org; accessed on 16 August 2024) is an internet portal and resource for the annotation and analysis of input gene sets [93], while the Molecular Complex Detection (MCODE) algorithm is used within Metascape to identify densely connected parts of the gene network, known as gene clusters [94]. In this research, MCODE analysis identified clusters among the 40 input genes. MCODE identifies groups of genes that physically interact with at least one other member of the input set. When this network includes 3 to 500 proteins, MCODE reveals highly interconnected elements of the network [93].

#### 4.2.4. Network Analysis: GeneMANIA, CytoNCA and CytoHubba

The online server GeneMANIA examines the interactions between the genes and enhances the input gene set by adding the most relevant connected genes [95]. In our study, this tool was used to explore interactions among the identified 40 mutual genes, focusing on the human organism (*H. sapiens*). The GeneMANIA tool can identify different types of gene interactions. Physical interactions refer to protein–protein interactions, where two gene products are linked based on evidence from protein studies. Co-expression relies on gene expression data, linking two genes if their expression patterns show similarity under various conditions. Genetic interactions denote functional connections between genes, often observed when a change in one gene affects the effect of another gene. Shared protein domains indicate that gene products share common protein domains. Co-localization denotes genes expressed in the same tissue or proteins localized at the same site within the cell. Interactions within molecular pathways indicate that two gene protein products participate in the same biochemical reaction within the molecular pathway [96]. Centrality analysis was conducted on the constructed GeneMania network to confirm the identified hub genes and identify additional key genes based on their connectivity and influence within the network. The network was analyzed by CytoNCA, a Cytoscape plug-in designed to calculate various centrality metrics such as degree, betweenness, and closeness. Degree centrality indicates the number of direct connections a gene has, betweenness centrality highlights genes that act as bridges within the network, and closeness centrality shows how quickly a gene can connect to others, thus identifying genes that are central to the network’s structure and communication [97]. Together, these metrics allowed for the identification of genes that may play central roles in the studied biological processes. The CytoHubba Cytoscape (3.10.3) tool was later employed to extract the top 5 genes within the constructed GeneMANIA network. For each PFAS chemical examined in the study, the relevant chemical–hub gene interaction data were extracted from the CTD. These interactions include various types of effects that chemicals can have on gene expression/mRNA expression.

**Figure 3:**
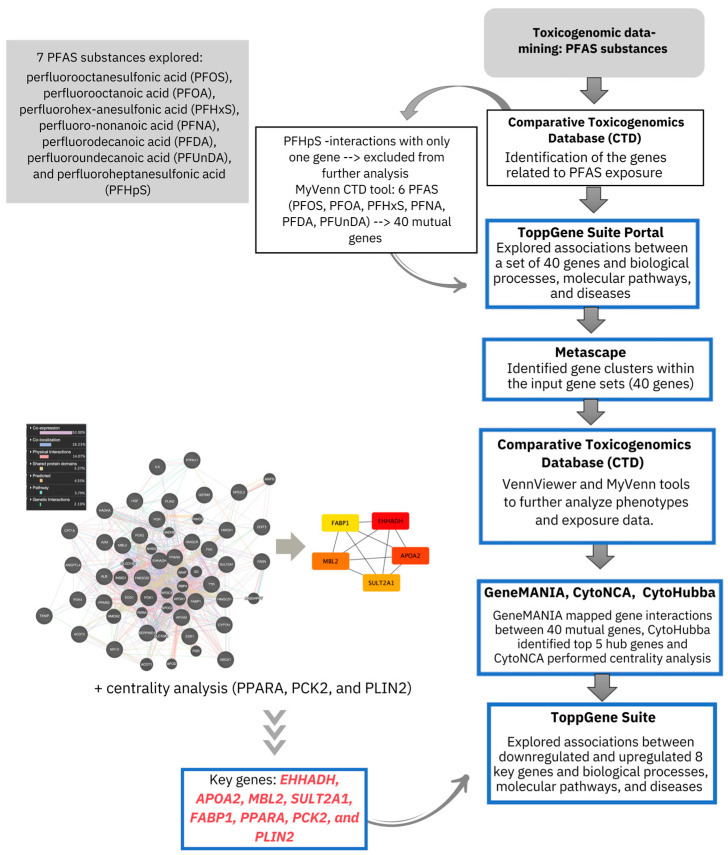
Flowchart illustrating the steps involved in the toxicogenomic analysis workflow.

A flowchart illustrating the steps involved in the toxicogenomic analysis workflow is presented in Figure 3.

## 5. Conclusions

The conducted analyses indicated that PFAS exhibit ADME properties that may influence their potential toxicity. These substances are potentially capable of intestinal absorption, passing through the blood–brain barrier, metabolic interferences, binding to estrogen receptors, and prolonged presence in the body, with implications for nephrotoxicity, CNS toxicity, eye and skin irritation, skin sensitization, and carcinogenicity. Forty genes common to all the investigated PFAS were identified, while *EHHADH*, *APOA2*, *MBL2*, *SULT2A1*, *FABP1*, *PPARA*, *PCK2*, and *PLIN2* were singled out as the most significant within this set. Each of these genes plays a different role in the organism, from beta-oxidation of fatty acids to cholesterol regulation, gluconeogenesis, and participation in the immune response. Phenotype data has shown that PFAS compounds impact cell death, growth, inflammation, steroid biosynthesis, and thyroid hormone metabolism, with effects varying based on the species, cell type, concentration, and co-exposure. Data from human biomonitoring studies has shown that, although fetal PFAS exposure was lower than in adults, these substances might still significantly influence metabolic processes, highlighting the need to monitor both adult and fetal exposure. The gene ontology and molecular pathway analysis of the shared gene set revealed the connections between PFAS exposure and processes mostly related to the disruption of lipid metabolism. Nuclear receptors and the PPAR signaling pathway, which are marked as important in MCODE analysis, are closely linked to these processes. These preliminary results strongly suggest the need for further in vitro and in vivo studies to confirm these interactions and their implications for human health, providing valuable direction for future research. Furthermore, the in-depth in silico analysis demonstrated in this study offers a valuable framework for gaining deeper insights into the molecular mechanisms behind the adverse effects induced not only by PFAS but also other chemical mixtures.

## Figures and Tables

**Table 1:** ADME-Tox prediction results for each of the examined PFAS (admetSAR; http://lmmd.ecust.edu.cn/admetsar2; accessed on 11 August 2024).

Category	PFOS	PFOA	PFHxS	PFNA	PFDA	PFUnDA
Absorption						
Crossing the blood–brain barrier	+	+	+	+	+	+
Intestinal absorption	+	+	+	+	+	+
Par 3 Caco-2	−	+	+	+	−	−
Bioavailability						
Oral bioavailability	+	−	+	−	−	−
Transporter inhibition						
The bile salt export pump (BSEP) inhibitor	−	−	−	−	−	+
Enzyme inhibition						
OATP1B1 inhibitor	−	−	+	+	+	+
OATP1B3 inhibitor	−	−	+	+	+	+
Receptor binding						
Aromatase binding	+	+	−	−	+	+
Binding of estrogen receptors	+	+	+	+	+	+
PPAR gamma	+	+	+	+	+	+
Toxicity						
Carcinogenicity (Total)	+	+	+	+	+	+
Eye damage	+	+	+	+	+	+
Eye irritation	+	+	+	+	+	+
Toxicity to fish	+	−	+	+	−	−
Micronuclearity	+	+	+	+	+	+
Mitochondrial toxicity	+	−	+	+	−	−
Nephrotoxicity	+	+	+	+	+	+
Reproductive toxicity	+	+	+	+	+	+
Respiratory toxicity	+	−	+	+	−	−
Skin damage	+	+	+	+	+	+
Skin irritation	+	+	+	+	+	+
Skin sensitization	+	+	+	+	+	+

A “+” indicates that the substance is associated with the respective ADMET property, while a “−” indicates no association according to the predictions.

**Table 2:** Molecular functions, biological processes, molecular pathways, and diseases associated with 40 genes common to all examined PFAS substances (ToppGene Suite; https://toppgene.cchmc.org; accessed on 16 August 2024).

	ID	Name	*p*-Value
Molecular functions	*GO:0004879*	activity of nuclear receptors	1.421 × 10^−6^
	*GO:0098531*	activity of a ligand-activated transcription factor	1.421 × 10^−6^
	*GO:0004421*	hydroxymethylglutaryl-CoA synthase activity	2.835 × 10^−6^
	*GO:0016509*	long-chain-3-hydroxyacyl-CoA dehydrogenase activity	8.481 × 10^−6^
	*GO:0003707*	activity of nuclear steroid receptors	1.789 × 10^−5^
	*GO:0008289*	lipid binding	3.732 × 10^−5^
	*GO:0046912*	acyltransferase activity, acyl groups are converted to alkyl during transfer	4.227 × 10^−5^
	*GO:0016491*	oxidoreductase activity	4.952 × 10^−5^
	*GO:0003857*	3-Hydroxyacyl-dehydrogenase activity	5.911 × 10^−5^
	*GO:0016616*	oxidoreductase activity, which acts on the CH-OH group of the donor, NAD or NADP as the acceptor	7.775 × 10^−5^
	*GO:0018812*	3-hydroxyacyl-CoA dehydratase activity	7.873 × 10^−5^
	*GO:0005496*	steroid binding	8.235 × 10^−5^
	*GO:0001221*	binding of transcription coregulators	9.473 × 10^−5^
	*GO:0016614*	oxidoreductase activity, which acts on the donor CH-OH group	1.028 × 10^−4^
	*GO:0001223*	binding of transcriptional coactivators	1.123 × 10^−4^
Biological processes	*GO:0006629*	lipid metabolism process	3.716 × 10^−21^
	*GO:0044255*	metabolic process of cellular lipids	3.531 × 10^−17^
	*GO:0031667*	response to nutrient levels	5.332 × 10^−15^
	*GO:0009991*	response to an extracellular stimulus	1.379 × 10^−14^
	*GO:0009410*	response to a xenobiotic stimulus	9.931 × 10^−14^
	*GO:0033993*	response to lipids	6.107 × 10^−13^
	*GO:0009725*	hormone response	3.006 × 10^−12^
	*GO:0014070*	response to an organic cyclic compound	5.254 × 10^−12^
	*GO:0008203*	metabolic process of cholesterol	5.852 × 10^−12^
	*GO:0006631*	metabolic process of fatty acids	5.858 × 10^−12^
	*GO:0016125*	metabolic process of sterols	1.142 × 10^−11^
	*GO:1902652*	secondary metabolic process of alcohol	1.323 × 10^−11^
	*GO:0008202*	steroid metabolic process	1.645 × 10^−11^
	*GO:1901701*	cellular response to an oxygen-containing compound	1.747 × 10^−11^
	*GO:0019395*	oxidation of fatty acids	2.288 × 10^−11^
Pathways	*M27316*	regulation of lipid metabolism by *PPARA*	5.093 × 10^−19^
	*M39428*	nuclear receptors	5.131 × 10^−18^
	*M39553*	PPAR signaling pathway	4.193 × 10^−16^
	*M13088*	PPAR signaling pathway	5.731 × 10^−16^
	*MM15995*	PPAR signaling pathway	1.041 × 10^−15^
	*M27451*	lipid metabolism	1.073 × 10^−14^
	*M39547*	*PPARA* road	3.066 × 10^−11^
	*MM15920*	cholesterol metabolism	3.457 × 10^−11^
	*M39679*	*SREBF* and *MIR33* in cholesterol and lipid homeostasis	5.509 × 10^−10^
	*M39853*	cholesterol metabolism	1.379 × 10^−9^
	*MM14563*	metabolism	3.431 × 10^−9^
	*MM15866*	nuclear receptors in lipid metabolism and toxicity	8.973 × 10^−9^
	*M41830*	cytoprotection by HMOX-1	9.346 × 10^−9^
	*MM15193*	lipid metabolism	1.027 × 10^−8^
	*M39488*	nuclear receptors in lipid metabolism	2.026 × 10^−8^
Diseases	*C0022661*	kidney failure, chronic	1.504 × 10^−14^
	*C0038433*	streptozotocin diabetes	1.150 × 10^−9^
	*C0011853*	diabetes mellitus, experimental	1.150 × 10^−9^
	*C0002152*	diabetes	1.150 × 10^−9^
	*C0860207*	drug-induced liver disease	1.669 × 10^−9^
	*C3658290*	acute drug-induced liver damage	1.669 × 10^−9^
	*C4277682*	liver damage caused by chemicals and drugs	1.669 × 10^−9^
	*C1262760*	drug-induced hepatitis	1.669 × 10^−9^
	*C0019193*	hepatitis, toxic	1.669 × 10^−9^
	*C4279912*	chemically induced hepatotoxicity	1.669 × 10^−9^
	*C0028754*	obesity	9.517 × 10^−9^
	*C1565662*	acute renal failure	2.269 × 10^−8^
	*C2609414*	acute kidney injury	2.269 × 10^−8^
	*C0022660*	kidney failure, acute	2.269 × 10^−8^
	*C1704377*	Bright’s disease	3.725 × 10^−8^

**Table 3:** Generated MCODE networks (depicted in Figure 1) and their associated biological pathways and processes (Metascape 3.5 software; https://metascape.org; accessed on 16 August 2024).

Color	MCODE	Gene Ontology	Description	Log10(P)
**Red**	MCODE_1	WP5434	Pathways in cancer	−9.8
	MCODE_1	hsa05200	Pathways in cancer	−9.7
	MCODE_1	WP3624	Lung fibrosis	−9.2
**Blue**	MCODE_2	WP2874	Liver X receptor pathway	−13.0
	MCODE_2	GO:0032787	Monocarboxylic acid metabolic process	−10.6
	MCODE_2	GO:0019752	Carboxylic acid metabolic process	−9.5
**Green**	MCODE_3	R-HSA-1989781	*PPARA* activates gene expression	−12.1
	MCODE_3	R-HSA-400206	Regulation of lipid metabolism by PPARalpha	−12.1
	MCODE_3	hsa03320	PPAR signaling pathway	−9.8

**Table 4:** Chemical–hub gene interactions and their effects on mRNA and protein expression for each examined PFAS chemical (Comparative Toxicogenomic Database (CTD) (https://ctdbase.org); Accessed on 16 August 2024).

	*EHHADH*		*APOA2*		*MBL2*		*SULT2A1*	
	ME	PE	ME	PE	ME	PE	ME	PE
PFOS	+	+	+/−	N/A	+/−	N/A	+/−	N/A
PFOA	+	+	+	+/−	+/−	N/A	+/−	−
PFHxS	+	N/A	+	N/A	+	N/A	+	N/A
PFNA	+/−	N/A	+	N/A	+	N/A	+/−	N/A
PFDA	+	N/A	+/−	N/A	−	N/A	−	N/A
PFUnDA	+	N/A	+/−	N/A	−	N/A	+	N/A
	*FABP1*		*PPARA*		*PCK2*		*PLIN2*	
	ME	PE	ME	PE	ME	PE	ME	PE
PFOS	+/−	N/A	+/−	N/A	+	N/A	+/−	N/A
PFOA	+/−	+	+/−	+/−	+	N/A	+/−	+
PFHxS	+	N/A	+/−	N/A	+	N/A	+	N/A
PFNA	+	N/A	+	N/A	+	N/A	+	N/A
PFDA	+	N/A	−	N/A	+	N/A	+	N/A
PFUnDA	−	N/A	+	N/A	+	N/A	+	N/A

ME: mRNA expression; PE: protein expression; +: increases; −: decreases; +/−: can either increase or decrease depending on the dose, species, duration of exposure, etc.

## Data Availability

These data were derived from the following resources available in the public domain: admetSAR (2.0) (https://lmmd.ecust.edu.cn/admetsar2; accessed on 11 August 2024), ADMETlab (2.0) (https://admetlab3.scbdd.com; accessed on 11 August 2024), Comparative Toxicogenomics Database (CTD; https://ctdbase.org; accessed on 15 August 2024), GeneMANIA (https://genemania.org; accessed on 15 August 2024 and 5 November 2024), CytoHubba (https://apps.cytoscape.org/apps/cytohubba; accessed on 15 August 2024) and CytoNCA (https://apps.cytoscape.org/apps/cytonca) Cytoscape (3.10.3) plug-ins (accessed on 15 August and 5 November 2024), ToppGene Suite portal (https://toppgene.cchmc.org; accessed on 16 August and 5 November 2024), and Metascape (3.5) (https://metascape.org; accessed on 16 August 2024).

## Supplementary Materials

The following supporting information can be downloaded at: https://www.mdpi.com/article/10.3390/ijms252212333/s1.
