# Supplementary material for: Exploring Toxicity of Per- and Polyfluoroalkyl Substances (PFAS) Mixture Through ADMET and Toxicogenomic In Silico Analysis: Molecular Insights
Source: Int J Mol Sci. 2024 Nov 17;25(22):12333. doi: 10.3390/ijms252212333 (PMC11594668; doi:10.3390/ijms252212333)
Supplement: Supplementary file 1 [file ijms-25-12333-s001.zip › Supplementary File S1.pdf]

Table S1. ADMET properties of the most prominent PFAS compounds ((ADMETlab (2.0); https://admetlab3.scbdd.com); accessed on 11 August 2024)

Property	PFOS	PFOA	PFHxS	*PFNA*	*PFDA*	*PFUnDA*
Molecular Weight (MW)	499.94	413.97	399.94	463.97	513.97	563.96
Volume	294.953	252.882	236.09	282.313	311.744	341.175
Density	1.695	1.637	1.694	1.643	1.649	1.653
logS	-1.894	-1.643	-1.316	-2.033	-2.357	-2.576
logP	1.817	2.061	1.476	2.427	2.708	2.864
logD7.4	1.302	1.797	1.175	2.026	2.192	2.282
pKa (Acid)	2.965	3.93	2.844	4.119	4.262	4.371
pKa (Base)	3.098	4.509	3.207	4.822	4.901	4.669
Melting point	99.0	60.285	89.35	69.404	77.843	86.142
Boiling point	248.721	185.511	233.389	200.342	213.097	222.185
Lipinski Rule	Accepted	Accepted	Accepted	Accepted	Accepted	Accepted
Pfizer Rule	Accepted	Accepted	Accepted	Accepted	Accepted	Accepted
GSK Rule	Rejected	Rejected	Accepted	Rejected	Rejected	Rejected
GoldenTriangle	Accepted	Accepted	Accepted	Accepted	Rejected	Rejected
PAINS	0	0	0	0	0	0
Alarm_NMR Rule	1	0	1	0	0	0
BMS Rule	0	0	0	0	1	1
Chelating Rule	0	0	0	0	0	0
PAMPA	+++	+++	+++	+++	+++	+++
Pgp inhibitor	+++	++	+++	+++	+++	+++
Pgp substrate	---	---	---	---	---	---
HIA	+++	---	+++	---	---	--
F20%	--	---	-	---	---	---
F30%	+++	---	+++	---	---	---
F50%	++	---	+	---	---	---
PPB	99.2%	99.1%	98.9%	99.1%	99.2%	99.3%
VDss	-0.007	-0.139	-0.099	-0.091	-0.044	-0.0
BBB	---	---	---	---	---	---
Fu	0.5%	0.6%	0.7%	0.5%	0.5%	0.4%
OATP1B1 inhibitor	---	---	---	---	---	---
OATP1B3 inhibitor	+++	++	+	+++	+++	+++
BCRP inhibitor	+++	+	+++	++	+++	+++
MRP1 inhibitor	+++	+++	+++	+++	+++	+++
BSEP inhibitor	+++	+++	++	+++	+++	+++
CYP1A2 inhibitor	---	---	---	---	---	---
CYP1A2 substrate	+++	+++	+++	+++	+++	+++
CYP2C19 inhibitor	---	+	---	++	+++	+++
CYP2C19 substrate	---	---	---	---	---	---
CYP2C9 inhibitor	+++	+	---	+++	+++	+++
CYP2C9 substrate	---	---	---	---	---	---
CYP2D6 inhibitor	---	---	---	---	---	---
CYP2D6 substrate	---	---	---	---	---	---
CYP3A4 inhibitor	---	---	---	---	---	---
CYP3A4 substrate	+++	+++	++	+++	+++	+++
CYP2B6 inhibitor	---	---	---	---	---	---
CYP2B6 substrate	---	---	---	---	---	---
CYP2C8 inhibitor	+++	+++	+++	+++	+++	+++
HLM Stability	---	---	---	---	---	---
CLplasma	2.412	1.91	2.473	1.869	1.837	1.799
T1/2	1.417	1.738	1.444	1.719	1.716	1.724
Acute Aquatic Toxicity Rule	1	2	1	2	2	2

Genotoxic Carcinogenicity Mutagenicity Rule	1	0	1	0	0	0
NonGenotoxic Carcinogenicity Rule	0	0	0	0	0	0
Skin Sensitization Rule	0	0	0	0	0	0
Aquatic Toxicity Rule	0	0	0	0	0	0
NonBiodegradable	1	1	1	1	1	1
SureChEMBL Rule	4	3	4	3	3	3
FAF-Drugs4 Rule	1	0	1	0	0	0
hERG Blockers	+	---	---	---	---	---
hERG Blockers (10um)	--	---	--	---	---	---
DILI	---	---	---	---	---	---
AMES Toxicity	--	--	--	--	--	--
Rat Oral Acute Toxicity	-	-	-	-	-	-
FDAMDD	-	--	-	--	--	--
Skin Sensitization	+	++	+	++	++	++
Carcinogenicity	-	-	-	-	-	-
Eye Corrosion	+++	+++	+++	+++	+++	+++
Eye Irritation	+++	+++	+++	+++	+++	+++
Respiratory	++	++	++	++	++	++
Human Hepatotoxicity	+	+	+	+	+	+
Drug-induced Nephrotoxicity	++	++	+	++	++	++
Drug-induced Neurotoxicity	--	--	--	--	---	---
Ototoxicity	+	-	-	-	+	+
Hematotoxicity	-	-	-	-	-	-
Genotoxicity	+++	+++	+++	+++	+++	+++
RPMI-8226 Immunitoxicity	---	---	---	---	---	---
A549 Cytotoxicity	---	---	---	---	---	---
Hek293 Cytotoxicity	---	---	---	---	---	---
BCF	3.178	2.621	2.996	2.726	2.83	2.931
IGC50	4.039	3.881	3.928	3.958	4.034	4.101
LC50DM	6.341	5.384	6.045	5.555	5.7	5.812
LC50FM	5.744	4.797	5.465	4.962	5.108	5.236
NR-AhR	---	---	---	---	---	---
NR-AR	--	---	--	---	---	---
NR-AR-LBD	---	---	---	---	---	---
NR-Aromatase	---	---	---	---	---	---
NR-ER	-	---	-	---	---	---
NR-ER-LBD	-	---	-	---	---	---
NR-PPAR-gamma	---	---	---	---	---	---
SR-ARE	-	+++	-	+++	+++	+++
SR-ATAD5	---	---	---	---	---	---
SR-HSE	-	--	-	--	--	--
SR-MMP	-	+	-	+	+	+
SR-p53	+	+++	-	+++	+++	+++

ADMET (Absorption, Distribution, Metabolism, Excretion, and Toxicity); Per- and polyfluoroalkyl substances (PFAS); Perfluorodecanoic acid (PFDA); Perfluorohexane sulfonic acid (PFHxS); Perfluorooctanoic acid (PFOA); Perfluorooctane sulfonate (PFOS); Perfluorononanoic acid (PFNA); Perfluoroundecanoic acid (PFUnDA). +++; ---: Represents a high or strong effect. ++; --: Indicates a moderate or medium effect. +; -: Suggests a low or weak effect.

Table S2. List of 40 mutual genes associated with exposure to the six examined PFAS compounds: Perfluorooctane sulfonate (PFOS), Perfluorooctanoic acid (PFOA), Perfluorohexane sulfonate (PFHxS), Perfluorononanoic acid (PFNA), Perfluorodecanoic acid (PFDA), and Perfluoroundecanoic acid (PFUnDA) (Comparative Toxicogenomics Database (CTD; https://ctdbase.org; accessed on 15 August 2024)).

GENE SYMBOL	GENE NAME
A2M	alpha-2-macroglobulin
ABCA1	ATP binding cassette subfamily A member 1
ACOT2	acyl-CoA thioesterase 2
ALB	albumin
ANGPTL4	angiopoietin like 4
APOA2	apolipoprotein A2
CPT1A	carnitine palmitoyltransferase 1A
CYP7A1	cytochrome P450 family 7 subfamily A member 1
DDIT3	DNA damage inducible transcript 3
EHHADH	enoyl-CoA hydratase and 3-hydroxyacyl CoA dehydrogenase
ESR1	estrogen receptor 1
FABP1	fatty acid binding protein 1
FAS	Fas cell surface death receptor
FASN	fatty acid synthase
GSTM3	glutathione S-transferase mu 3
HADHA	hydroxyacyl-CoA dehydrogenase trifunctional multienzyme complex subunit alpha
HGF	hepatocyte growth factor
HMGCR	3-hydroxy-3-methylglutaryl-CoA reductase
HMGCS1	3-hydroxy-3-methylglutaryl-CoA synthase 1
HMGCS2	3-hydroxy-3-methylglutaryl-CoA synthase 2
HMOX1	heme oxygenase 1
IL6	interleukin 6
MBL2	mannose binding lectin 2
NFE2L2	NFE2 like bZIP transcription factor 2
NR1I2	nuclear receptor subfamily 1 group I member 2
PCK2	phosphoenolpyruvate carboxykinase 2, mitochondrial
PDK4	pyruvate dehydrogenase kinase 4
PLIN2	perilipin 2
POR	cytochrome p450 oxidoreductase
PPARA	peroxisome proliferator activated receptor alpha
PPARG	peroxisome proliferator activated receptor gamma
PTPN11	protein tyrosine phosphatase non-receptor type 11
SERPIND1	serpin family D member 1
SOD1	superoxide dismutase 1
SULT2A1	sulfotransferase family 2A member 1
TTR	transthyretin
TXNIP	thioredoxin interacting protein

Centrality analysis was conducted on the GeneMania network using CytoNCA (available at https://apps.cytoscape.org/apps/cytonca; accessed on 5 November 2024). The table presents the centrality metrics, highlighting key genes based on degree, betweenness, and closeness centrality measures. These results provide insight into the potential roles of genes within the network structure and their importance in biological processes.

Table S3. Centrality Analysis Results for the GeneMania Network, sorted by degree. Top scores for each centrality measure (degree, betweenness, closeness) are highlighted in orange CytoNCA (available at https://apps.cytoscape.org/apps/cytonca; accessed on 5 November 2024).

GENE NAME	DEGREE	CLOSENESS	BETWEENNESS
FABP1	32.00	0.67	63.46
EHHADH	30.00	0.68	61.06
APOA2	28.00	0.67	65.36
PPARA	27.00	0.64	52.58
PCK2	24.00	0.71	111.04
MBL2	23.00	0.62	25.86
HMGCS2	20.00	0.61	17.27
PLIN2	19.00	0.63	75.18
PPARG	18.00	0.60	26.98
SULT2A1	18.00	0.60	19.81
SERPIND1	18.00	0.60	17.04
HMGCS1	18.00	0.59	28.96
TTR	17.00	0.59	14.94
SOD1	16.00	0.62	52.49
POR	16.00	0.62	44.04
ALB	16.00	0.55	10.06
ANGPTL4	15.00	0.57	38.72
NR1I2	14.00	0.59	35.98
HGF	13.00	0.59	31.24
CYP7A1	13.00	0.57	33.40
ESR1	13.00	0.56	8.44
FAS	12.00	0.59	42.74
HMOX1	12.00	0.54	26.04
PDK4	11.00	0.56	31.42
HADHA	11.00	0.56	6.76
HMGCR	11.00	0.55	12.50
CPT1A	9.00	0.52	2.76
A2M	8.00	0.55	26.40
PTPN11	8.00	0.49	17.86
ABCA1	7.00	0.51	1.55
ACOT2	6.00	0.51	1.33
GSTM3	5.00	0.52	7.32
DDIT3	5.00	0.49	2.24

NFE2L2	5.00	0.47	4.49
IL6	5.00	0.47	3.44
TXNIP	5.00	0.43	1.40
FASN	4.00	0.49	5.85

The following tables contain extracted ToppGene enrichment results (molecular functions, biological processes, pathways and diseases) for the following key genes:

Genes upregulated by PFAS: *EHHADH, APOA2, FABP1, PPARA, PCK2*, and *PLIN2*.

Table S4. ToppGene enrichment results (molecular functions, biological processes, pathways and diseases) for genes upregulated by per- and polyfluoroalkyl substances (PFAS): *EHHADH, APOA2, FABP1, PPARA, PCK2*, and *PLIN2* (ToppGene Suite portal ((https://toppgene.cchmc.org; accessed on 5 November 2024))

1: GO: Molecular Function [Display Chart] 6 input genes in category / 121 annotations before applied cutoff / 19912 genes in category

	ID	Name	Source	pValue	FDR B&H	FDR B&Y	Bonferroni	Genes from Input	Genes in Annotation
1	GO:0051978	lysophospholipid:sodium symporter activity		6.026E-4	1.943E-2	1.045E-1	7.291E-2	1	2
2	GO:0004611	phosphoenolpyruvate carboxykinase activity		6.026E-4	1.943E-2	1.045E-1	7.291E-2	1	2
3	GO:0004613	phosphoenolpyruvate carboxykinase (GTP) activity		6.026E-4	1.943E-2	1.045E-1	7.291E-2	1	2
4	GO:0016509	long-chain-3-hydroxyacyl-CoA dehydrogenase activity		9.038E-4	1.943E-2	1.045E-1	1.094E-1	1	3
5	GO:0070653	high-density lipoprotein particle receptor binding		9.038E-4	1.943E-2	1.045E-1	1.094E-1	1	3

Show 45 more annotations

2: GO: Biological Process [Display Chart] 6 input genes in category / 642 annotations before applied cutoff / 20649 genes in category

	ID	Name	Source	pValue	FDR B&H	FDR B&Y	Bonferroni	Genes from Input	Genes in Annotation
1	GO:0044242	cellular lipid catabolic process		1.706E-9	1.096E-6	7.715E-6	1.096E-6	5	257
2	GO:0016042	lipid catabolic process		1.332E-8	3.451E-6	2.430E-5	8.550E-6	5	387
3	GO:0009062	fatty acid catabolic process		1.613E-8	3.451E-6	2.430E-5	1.035E-5	4	120
4	GO:0072329	monocarboxylic acid catabolic process		3.968E-8	6.368E-6	4.485E-5	2.547E-5	4	150
5	GO:0050996	positive regulation of lipid catabolic process		1.341E-7	1.722E-5	1.213E-4	8.612E-5	3	40

Show 45 more annotations

3: GO: Cellular Component [Display Chart] 6 input genes in category / 34 annotations before applied cutoff / 20915 genes in category

	ID	Name	Source	pValue	FDR B&H	FDR B&Y	Bonferroni	Genes from Input	Genes in Annotation
1	GO:0031907	microbody lumen		1.087E-4	1.848E-3	7.610E-3	3.696E-3	2	57
2	GO:0005782	peroxisomal matrix		1.087E-4	1.848E-3	7.610E-3	3.696E-3	2	57

3	GO:0005777	peroxisome		9.308E-4	7.912E-3	3.258E-2	3.165E-2	2	167
4	GO:0042579	microbody		9.308E-4	7.912E-3	3.258E-2	3.165E-2	2	167
5	GO:0034366	spherical high-density lipoprotein particle		2.579E-3	1.670E-2	6.877E-2	8.770E-2	1	9

Show 9 more annotations

4: Pathway [Display Chart] 6 input genes in category / 214 annotations before applied cutoff / 13850 genes in category

	ID	Name	Source	pValue	FDR B&H	FDR B&Y	Bonferroni	Genes from Input	Genes in Annotation
1	M39553	WP PPAR SIGNALING PATHWAY	WikiPathways	1.360E-11	1.509E-9	8.970E-9	2.911E-9	5	67
2	M13088	KEGG PPAR SIGNALING PATHWAY	KEGG Legacy Pathways	1.583E-11	1.509E-9	8.970E-9	3.387E-9	5	69
3	MM15995	WP PPAR SIGNALING PATHWAY	WikiPathways	2.115E-11	1.509E-9	8.970E-9	4.526E-9	5	73
4	M39547	WP PPAR ALPHA PATHWAY	WikiPathways	1.460E-10	7.809E-9	4.643E-8	3.124E-8	4	26
5	M2404	BIOCARTA PPARA PATHWAY	BioCarta Pathways	2.635E-9	1.128E-7	6.706E-7	5.639E-7	4	52

Show 45 more annotations

5: Disease [Display Chart] 6 input genes in category / 238 annotations before applied cutoff / 29490 genes in category

	ID	Name	Source	pValue	FDR B&H	FDR B&Y	Bonferroni	Genes from Input	Genes in Annotation
1	C0022672	Acute Kidney Tubular Necrosis	DisGeNET Curated	2.069E-7	4.925E-5	2.981E-4	4.925E-5	2	4
2	C0022661	Kidney Failure, Chronic	DisGeNET Curated	3.875E-5	3.459E-3	2.093E-2	9.223E-3	2	48
3	C0015695	Fatty Liver	DisGeNET Curated	1.310E-4	3.459E-3	2.093E-2	3.119E-2	2	88
4	C2711227	Steatohepatitis	DisGeNET Curated	1.310E-4	3.459E-3	2.093E-2	3.119E-2	2	88
5	DOID:9427 (implicated via orthology)	hypertensive encephalopathy (implicated via orthology)	AllianceGenome	2.035E-4	3.459E-3	2.093E-2	4.842E-2	1	1

Show 45 more annotations

Genes downregulated by PFAS: *MBL2, SULT2A1, FABP1, PPARA* (downregulated by PFDA and/or PFUnDa

Table S5. ToppGene enrichment results (molecular functions, biological processes, pathways and diseases) for genes downregylated by per- and polyfluoroalkyl substances (PFAS): *MBL2, SULT2A1, FABP1, PPARA* (downregulated by perfluorodecanoic acid (PFDA) and/or perfluoroundecanoic acid (PFUnDa)) ToppGene Suite portal ((https://toppgene.cchmc.org; accessed on 5 November 2024))

1: GO: Molecular Function [Display Chart] 4 input genes in category / 93 annotations before applied cutoff / 19912 genes in category

	ID	Name	Source	pValue	FDR B&H	FDR B&Y	Bonferroni	Genes from Input	Genes in Annotation
1	GO:0033876	glycochenodeoxycholate sulfotransferase activity		2.009E-4	1.401E-2	7.166E-2	1.868E-2	1	1
2	GO:0051978	lysophospholipid:sodium symporter activity		4.017E-4	1.401E-2	7.166E-2	3.736E-2	1	2
3	GO:0008289	lipid binding		5.097E-4	1.401E-2	7.166E-2	4.740E-2	3	1016
4	GO:0047704	bile-salt sulfotransferase activity		6.026E-4	1.401E-2	7.166E-2	5.604E-2	1	3
5	GO:0004027	alcohol sulfotransferase activity		8.034E-4	1.494E-2	7.643E-2	7.471E-2	1	4

Show 34 more annotations

2: GO: Biological Process [Display Chart] 4 input genes in category / 511 annotations before applied cutoff / 20649 genes in category

	ID	Name	Source	pValue	FDR B&H	FDR B&Y	Bonferroni	Genes from Input	Genes in Annotation
1	GO:0072329	monocarboxylic acid catabolic process		1.495E-6	5.605E-4	3.820E-3	7.639E-4	3	150
2	GO:0032000	positive regulation of fatty acid beta-oxidation		2.194E-6	5.605E-4	3.820E-3	1.121E-3	2	13
3	GO:0046321	positive regulation of fatty acid oxidation		7.111E-6	8.409E-4	5.731E-3	3.634E-3	2	23
4	GO:0031998	regulation of fatty acid beta-oxidation		9.133E-6	8.409E-4	5.731E-3	4.667E-3	2	26
5	GO:0046395	carboxylic acid catabolic process		9.874E-6	8.409E-4	5.731E-3	5.046E-3	3	281

Show 45 more annotations

3: Pathway [Display Chart] 4 input genes in category / 128 annotations before applied cutoff / 13850 genes in category

	ID	Name	Source	pValue	FDR B&H	FDR B&Y	Bonferroni	Genes from Input	Genes in Annotation
1	M27316	REACTOME REGULATION OF LIPID METABOLISM BY PPARALPHA	Reactome Pathways	2.397E-6	3.068E-4	1.667E-3	3.068E-4	3	118
2	MM15034	REACTOME REGULATION OF LIPID METABOLISM BY PPARALPHA	Reactome Pathways	9.557E-6	6.117E-4	3.323E-3	1.223E-3	2	18

3	M39547	WP PPAR ALPHA PATHWAY	WikiPathways	2.029E-5	8.655E-4	4.703E-3	2.597E-3	2	26
4	M39428	WP NUCLEAR RECEPTORS META PATHWAY	WikiPathways	4.540E-5	1.453E-3	7.893E-3	5.811E-3	3	314
5	M2404	BIOCARTA PPARA PATHWAY	BioCarta Pathways	8.256E-5	1.900E-3	1.032E-2	1.057E-2	2	52

Show 45 more annotations

4: Disease [Display Chart] 4 input genes in category / 302 annotations before applied cutoff / 29490 genes in category

	ID	Name	Source	pValue	FDR B&H	FDR B&Y	Bonferroni	Genes from Input	Genes in Annotation
1	C0022672	Acute Kidney Tubular Necrosis	DisGeNET Curated	8.279E-8	2.500E-5	1.572E-4	2.500E-5	2	4
2	C0022661	Kidney Failure, Chronic	DisGeNET Curated	1.553E-5	2.048E-3	1.288E-2	4.691E-3	2	48
3	DOID:1485 (is marker for)	cystic fibrosis (is marker for)	AllianceGenome	2.602E-5	2.048E-3	1.288E-2	7.859E-3	2	62
4	DOID:10652 (is implicated in)	Alzheimer's disease (is implicated in)	AllianceGenome	1.168E-4	2.048E-3	1.288E-2	3.528E-2	2	131
5	C3280586	Mannose-Binding Protein Deficiency	DisGeNET Curated	1.356E-4	2.048E-3	1.288E-2	4.096E-2	1	1

Show 45 more annotations

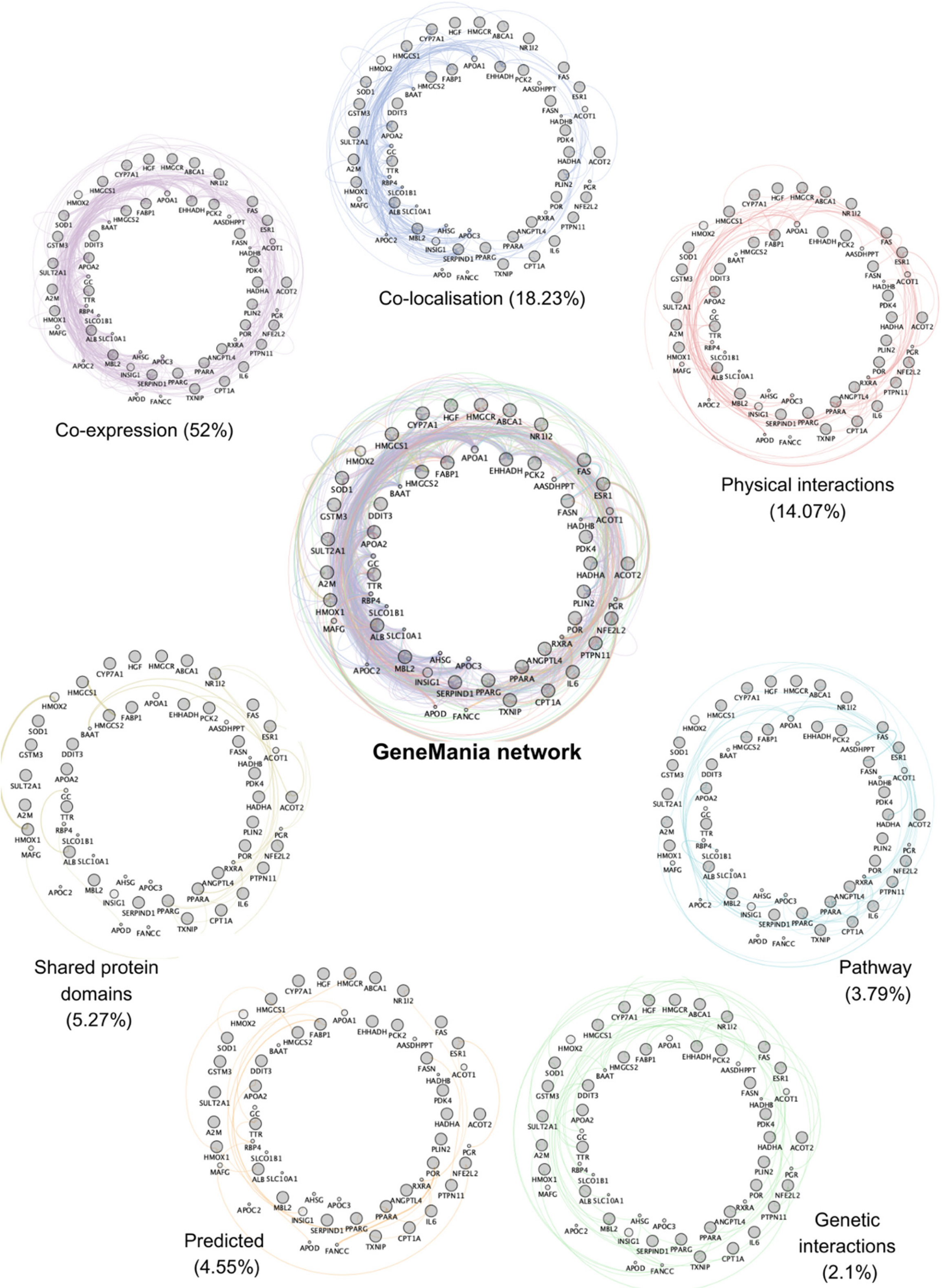

Figure S1. GeneMania network in circular layout: combined and interaction-specific views (GeneMANIA (https://genemania.org; accessed on 5 November 2024))
